# Characterization of LysB4, an endolysin from the *Bacillus cereus*-infecting bacteriophage B4

**DOI:** 10.1186/1471-2180-12-33

**Published:** 2012-03-15

**Authors:** Bokyung Son, Jiae Yun, Jeong-A Lim, Hakdong Shin, Sunggi Heu, Sangryeol Ryu

**Affiliations:** 1 Department of Food and Animal Biotechnology, Seoul National University, 599 Gwanak-ro, Gwanak-gu, Seoul 151-921, Republic of Korea; 2 Department of Agricultural Biotechnology, Center for Agricultural Biomaterials, and Research Institute for Agriculture and Life Sciences, Seoul National University, Seoul 151-921, South Korea; 3 Microbial Safety Division, National Academy of Agricultural Science, Rural Development Administration, Suwon 441-707, South Korea

## Abstract

**Background:**

*Bacillus cereus *is a foodborne pathogen that causes emetic or diarrheal types of food poisoning. The incidence of *B. cereus *food poisoning has been gradually increasing over the past few years, therefore, biocontrol agents effective against *B. cereus *need to be developed. Endolysins are phage-encoded bacterial peptidoglycan hydrolases and have received considerable attention as promising antibacterial agents.

**Results:**

The endolysin from *B. cereus *phage B4, designated LysB4, was identified and characterized. *In silico *analysis revealed that this endolysin had the VanY domain at the N terminus as the catalytic domain, and the SH3_5 domain at the C terminus that appears to be the cell wall binding domain. Biochemical characterization of LysB4 enzymatic activity showed that it had optimal peptidoglycan hydrolase activity at pH 8.0-10.0 and 50°C. The lytic activity was dependent on divalent metal ions, especially Zn^2+^. The antimicrobial spectrum was relatively broad because LysB4 lysed Gram-positive bacteria such as *B. cereus, Bacillus subtilis *and *Listeria monocytogenes *and some Gram-negative bacteria when treated with EDTA. LC-MS analysis of the cell wall cleavage products showed that LysB4 was an L-alanoyl-D-glutamate endopeptidase, making LysB4 the first characterized endopeptidase of this type to target *B. cereus*.

**Conclusions:**

LysB4 is believed to be the first reported L-alanoyl-D-glutamate endopeptidase from *B. cereus*-infecting bacteriophages. The properties of LysB4 showed that this endolysin has strong lytic activity against a broad range of pathogenic bacteria, which makes LysB4 a good candidate as a biocontrol agent against *B. cereus *and other pathogenic bacteria.

## Background

*Bacillus cereus *is a Gram-positive, spore-forming, rod-shape bacterium that grows well in aerobic and anaerobic environments [1]. It causes food poisoning by producing two different types of toxins: an emetic toxin and a diarrheal toxin [2]. Although the symptoms caused by *B. cereus *food poisoning are relatively mild, the incidence of the disease is gradually increasing, and it can develop into severe disease [3]. In addition, *B. cereus *can survive at a wide temperature range and form spores in harsh environments, especially during food processing; therefore, measures to control *B. cereus *effectively in the food industry are necessary [4,5].

Recently, endolysins have been explored as promising antibacterial agents. Endolysins are phage-encoded enzymes that hydrolyze the peptidoglycan bacterial cell wall [6]. Endolysins are synthesized at the end of the phage replication cycle and allow liberation of progeny phage particles from the host cell [7]. Most endolysins lack secretory signal sequences, therefore, holins are needed for endolysins to pass through the inner membrane and reach peptidoglycan, defined as the canonical holin-endolysin lysis system [6,8].

Endolysins are expected to be more effective biocontrol agents toward Gram-positive than Gram-negative bacteria, because the latter have an outer membrane that blocks access of endolysins to the peptidoglycan layer, when applied exogenously [9]. In addition, other advantages of endolysins as biocontrol agents include: (i) low chance of developing bacterial resistance; (ii) species-specific lytic activity without affecting other bacteria; and (iii) high enzymatic activity that enables bacterial cells lysis within minutes or even seconds [7,10,11]. Endolysins are successfully applied in food products, such as milk and banana juice, to prevent contamination of *Staphylococcus aureus *or Gram-negative bacteria [12,13]. Besides, many reports already have shown that endolysins have high potential as strong therapeutic agents against a number of human pathogens through animal model studies [7,14-16].

To date, only three endolysins from *B. cereus *bacteriophages have been characterized, all of which are N-acetylmuramoyl-L-alanine amidase-type endolysins [17]. Moreover, only a few reported phages can infect *B. cereus*, although many *Bacillus*-targeting bacteriophages have been reported [18,19]. Thus, more bacteriophages and endolysins targeting *B. cereus *should be isolated and characterized to provide additional candidates for *B. cereus *biocontrol agents.

In previous work, we isolated the bacteriophage B4 (accession no. JN790865), which is a lytic phage infecting *B. cereus*, from forest mud, and its genome was sequenced and analyzed to annotate important features (Shin *et al.*, unpublished). In the present study, an endolysin gene was identified in the B4 bacteriophage genome. This endolysin gene was cloned and expressed in *Escherichia coli*, and the purified endolysin was characterized for its biochemical properties. To the best of our knowledge, LysB4 is the first endolysin belonging to the L-alanoyl-D-glutamate endopeptidases originating from *B. cereus *bacteriophages.

## Results

### Identification and expression of the LysB4 phage endolysin

**Figure 1:**
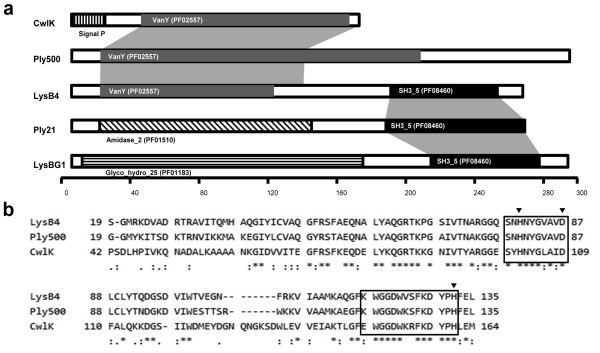
**Sequence analysis of LysB4**. **(a) **Domain structures of LysB4 compared with four other peptidoglycan hydrolases. CwlK, the cell wall hydrolase in *B. subtilis *(ZP 08507241); Ply500, an endolysin in a *L. monocytogenes *phage (CAA59365); Ply21, an endolysin in a *B. cereus *phage (CAA72267); and LysBG1, an endolysin from a *Bacillus *phage (ABX56141). The grey shadows indicate conserved regions between proteins. **(b) **Alignment of amino acid sequences of LysB4, Ply500 and CwlK in their VanY domains. Three small triangles indicate Zn^2+ ^binding residues, and the zinc binding motif was boxed.

Annotation of bacteriophage B4 genome sequence identified a predicted open reading frame (ORF) for a putative endolysin gene (Shin *et al.*, unpublished). This 789-bp-long ORF was designated *lysB4*. Using the InterProScan program (http://www.ebi.ac.uk/Tools/pfa/iprscan/), LysB4 was predicted to have the VanY domain (PF02557) at the N terminus and SH3_5 domain (PF08460) at the C terminus (Figure 1a). According to BLASTP analysis [20], the N terminus of LysB4 had high similarity to L-alanoyl-D-glutamate peptidases of *Listeria monocytogenes *FSL J1-175 (ZP 05387674*), Bacillus subtilis *subsp. subtilis str. 168 (CwlK, NP 388163), the *Listeria *phage A500 (Ply500, YP 001488411) and the *Bacillus *phage SPO1 (YP 001487954), and the C terminus had high similarity to proteins belonging to *B. cereus *AH676 (ZP 0419059)*, Bacillus *phages TP21-L (Ply21, CAA72267) and bg1 (LysBG1, ABX56141), and the *Lactobacillus *phage LL-Ku (AAV30211). Among these proteins, Ply500 of *Listeria *phage A500 needs Zn^2+ ^in its active site according to a structural analysis [21]. The three Zn^2+^-coordinating residues (His80, Asp87 and His133) characterized in PlyA500 were conserved in the amino acid sequence of LysB4 [21], and the Zn^2+ ^binding domain (SxHxxGxAxD) reported in *Enterococcus *VanX was found in LysB4 (Figure 1b) [22].

**Figure 2:**
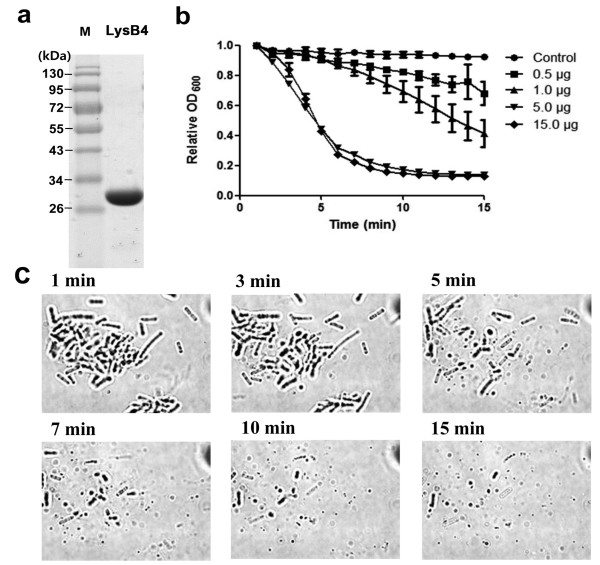
**Purification of LysB4 and lytic activity of LysB4. (a) **Purified LysB4 was loaded on an SDS-PAGE gel. Lane M, molecular weight marker; lane 1, the purified LysB4 fraction. **(b) **Different concentration of LysB4 was added to the suspension of *B.cereus *ATCC 10876, and decrease in turbidity was monitored. **(c) **Diluted suspension of *B. cereus *ATCC10876 (100 μl) was mixed with 5 μg of LysB4 and observed under optical microscope (× 1,000 magnification).

Recombinant LysB4 was cloned and expressed in *E. coli *with an N-terminal His-tag followed by purification using affinity chromatography. SDS-PAGE showed a single band between 26 and 34 kDa, which was consistent with the calculated molecular mass (28 kDa; Figure 2a). Only 5 μg of purified LysB4 could lyse *B. cereus *ATCC 10876 cells substantially in 5 min (Figure 2b). Viable cell counting revealed that 5 μg of LysB4 under this reaction condition could reduce the viable cell number by 3 to 4-log after 15 min (data not shown). Moreover, typical optical microscopy showed that most bacilli were ruptured and disappeared by addition of LysB4 within 15 min (Figure 2c).

### Effect of pH, temperature and ionic strength

**Figure 3:**
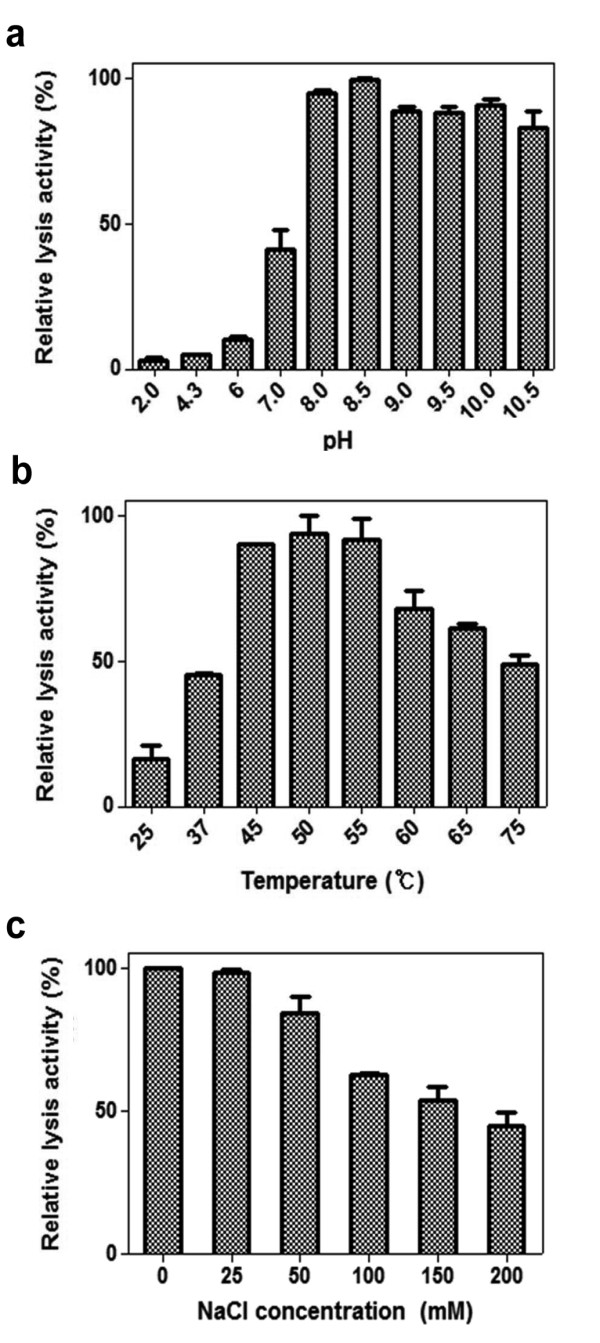
**Effect of pH, temperature, and NaCl on the lytic activity of LysB4**. The effect of pH **(a)**, temperature **(b)**, and NaCl concentration **(C) **on the lytic activity of LysB4 against *B. cereus *ATCC 10876 cells was shown. Relative lytic activity was obtained by comparing the lytic activity of each test with the maximal lytic activity among the dataset. Each column represents the mean of triplicate experiments, and error bars indicate the standard deviation.

Analysis of lytic activity at different pH showed that LysB4 had the highest lytic activity at pH 8.0-10.0 (Figure 3a). This endolysin was relatively stable under a wide range of pH values, as incubation at pH 2.0-10.5 for 30 min did not inactivate the lytic activity (data not shown). In addition, although this endolysin was active to lyse the susceptible bacteria between 37 and 75°C, the maximal activity was shown at 50°C (Figure 3b). However, LysB4 was inactivated when it was incubated at > 55°C for 30 min (data not shown). The influence of NaCl on the lytic activity of LysB4 was determined from 0-200 mM NaCl. As the NaCl concentrations increased, LysB4 lytic activity was reduced, resulting in approximately 60% decrease in the presence of 200 mM NaCl (Figure 3c).

### Effect of divalent metal ions

To examine the effects of divalent metal ions to LysB4 enzymatic activity, we first removed metal ions from the protein using 5.0 mM EDTA. As seen in Table 1 incubation of endolysin with 5 mM EDTA significantly decreased the lytic activity, which suggests LysB4 required metal ions for its full lytic activity. When 0.1 mM Zn^2+^or Mn^2+ ^was added to the EDTA-treated endolysin, the lytic activity of the enzyme was restored (Table 1). In the case of other divalent metal ions, such as Ca^2+ ^and Mg^2+^, addition of higher concentration (1 mM) restored LysB4 enzymatic activity. However, addition of Hg^2+ ^and Cu^2+ ^did not resort activity of the EDTA-treated endolysin. Taken together, LysB4 requires divalent metal ions, particularly Zn^2+ ^or Mn^2+ ^for its enzymatic activity.

**Table 1:** Effect of metal ions on lytic activity of EDTA-treated LysB4

	Relative lytic activity (%)	
Untreated	100	
5 mM EDTA	8.5 ± 0.2	
Metal ions	0.1 mM	1.0 mM
Zn^2+^	104 ± 2.8	Not available
Mn^2+^	89.5 ± 17.6	96 ± 8.4
Ca^2+^	34.5 ± 12.0	90 ± 11.3
Mg^2+^	32 ± 9.8	90.2 ± 9.6
Hg^2+^	8.3 ± 2.5	Not available
Cu^2+^	17.2 ± 5.9	12.5 ± 0.7

Relative lytic activities were measured by comparing the lytic activity of tests with it of LysB4 that was not treated with EDTA initially (Untreated). Values represent the mean ± standard deviation (n = 3).

### Antimicrobial spectrum of LysB4

Antimicrobial activity against several Gram-positive and Gram-negative bacteria (Table 2) was examined. Six *B. cereus *strains, *B. subtilis*, and two *L. monocytogenes *strains were susceptible to 5 μg LysB4, showing complete lysis in the reaction buffer within 5 min. This enzyme did not show lytic activity against other Gram-positive bacteria such as *Enterococcus faecalis, Staphylococcus aureus *strains, *Streptococcus thermophilus and Lactococcus lactis*. Furthermore, LysB4 lytic activity was not detected with Gram-negative bacteria, since they have a different cell wall composition (e.g., outer membrane) from Gram-positive bacteria. However, when cells were washed with 0.1 M EDTA to increase the cell wall permeability, LysB4-mediated cell lysis was detected for all tested Gram-negative bacteria including *E. coli, Pseudomonas aeruginosa, Cronobacter sakazakii, Salmonella *Typhimurium strains, *Salmonella *Enteritidis, *Shigella flexneri*, and *Shigella boydii*. In particular, *E. coli *O157:H7 strains were lysed efficiently by LysB4.

**Table 2:** The antimicrobial spectrum of LysB4

Organisms		Relative lytic activity (%)
Gram-negative bacteria	*Escherichia coli *MG1655	++
	*Escherichia coli *O157:H7 ATCC 43894	++
	*Escherichia coli *O157:H7 ATCC 43890	++
	*Escherichia *coli O157:NM 3204-92	++
	*Pseudomonas aeruginosa *ATCC 27853	++
	*Cronobacter sakazakii *ATCC 29544	++
	*Shigella flexineri *2a strain 2457 T	+
	*Shigella boydii *IB 2474	++
	*Salmonella *Typhimurium LT2	+
	*Salmonella *Enteritidis ATCC 13078	+
Gram-positive bacteria	*Listeria monocytogenes *ATCC 19114	++
	*Bacillus cereus *ATCC 40133	+++
	*Bacillus cereus *ATCC 27348	+++
	*Bacillus subtilis *168	+++
	*Enterococcus faecalis *ATCC 29212	-
	*Staphylococcus aureus *ATCC 29213	-
	*Lactococcus lactis *subsp. Lactis ATCC 11454	-
	*Streptococcus thermophilus *ATCC 19258	-

Gram-negative bacteria were treated with EDTA. Relative lytic activity was obtained by comparing the lytic activity of each test to it toward *B. cereus *ATCC10876; 1-40% +, 41-70% ++, 71-100% +++, 0% -

### Endopeptidase activity of LysB4

LysB4 had the VanY domain at its N terminus. The VanY domain encoded an L-alanoyl-D-glutamate endopeptidase and therefore LysB4 was expected to have endopeptidase activity. This was confirmed using the trinitrobenzene sulfonic acid (TNBS) method that detects the liberated free amino groups from *B. cereus *peptidoglycan caused by hydrolysis of LysB4. Pre-existing amino groups were eliminated by acetylating the peptidoglycan. We detected a high concentration of free amino groups (0.37 mM) released from the acetylated peptidoglycan after incubation with LysB4 (15 μg) for 1 h, whereas only 0.04 mM was released from the peptidoglycan in the absence of LysB4. Moreover, this enzyme did not show any N-acetylmuramoyl-L-alanine amidase or glycosidase activity (data not shown). Therefore, LysB4 belongs to the endopeptidases.

### Determination of the cleavage site by LysB4 in the peptidoglycan

**Figure 4:**
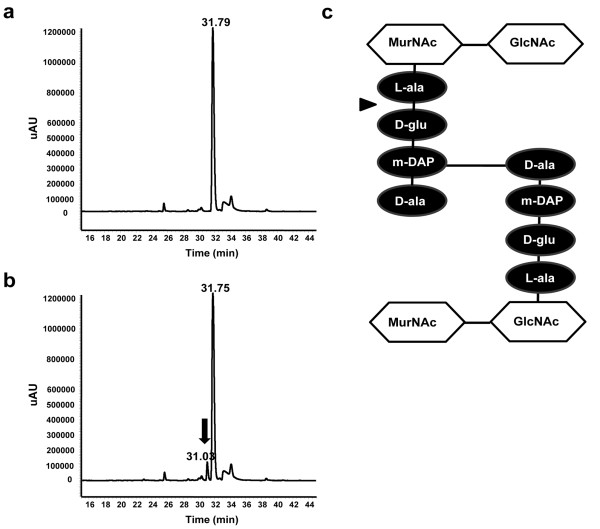
**LysB4 cleavage site in peptidoglycan**. **(a, b) **HPLC results with the enzymatic reaction products of LysB4. Purified cell wall of *B. cereus *was reacted with LysB4 for 0 min **(a) **and 60 min **(b)**. **(c) **Structure of peptidoglycan in *Bacillus *species. The cleavage site by the LysB4 was indicated by an arrow.

The specific LysB4 cleavage site in the peptidoglycan was determined by reverse-phase (RP)-HPLC and LC-MS (Figure 4). A peak that was absent from the control reaction (Figure 4a) and had a retention time of 31.03 min was observed in cell wall samples digested with LysB4 (arrow, Figure 4b). This peak corresponded to a fragment ion at m/z of 311.86, which seemed to be the [M-H]^- ^of 2,4-dinitrophenol (DNP)-D-Glu (*M*r, 313). Both peaks at 31.75 min in Figure 4a and at 31.79 min in Figure 4b corresponded to DNP. When non-acetylated and acetylated peptidoglycan substrate were hydrolyzed by 4 N HCl and analyzed by RP-HPLC, the peak corresponding to DNP-D-diaminopimelic acid (*M*r, 355) appeared only with the non-acetylated peptidoglycan sample, which showed that free amino groups of diaminopimelic acid in non-cross-linked peptide stem were labeled with DNP in this sample (data not shown). The lack of this peak with the acetylated peptidoglycan sample indicated that all the free amino groups were successfully acetylated. These results suggested that LysB4 acts as an L-alanoyl-D-glutamate endopeptidase to cut the peptide bond between the L-Ala and D-Glu (arrow, Figure 4c).

## Discussion

In this study, LysB4, a newly identified endolysin from the *B. cereus*-specific bacteriophage B4, was expressed, purified, and characterized. We showed that LysB4 was an L-alanoyl-D-glutamate endopeptidase. These endopeptidases have been reported in *L. monocytogenes *phages, the *E. coli *bacteriophage T5, and a *B. subtilis *strain [21,23,24]. In contrast, all the characterized endolysins found in bacteriophages infecting *Bacillus *species are amidases (Ply21, Ply12, and PlyBa) [17]. Thus, LysB4 is the first characterized L-alanoyl-D-glutamate endopeptidase originating from *B. cereus *phages.

LysB4 has two domains; the VanY domain at the N-terminus and SH3_5 domain at the C-terminus. The majority of the endolysins have two domains connected by a short linker: the N-terminal catalytic domain is responsible for cell lytic activity and the C-terminal cell wall binding domain that recognizes and binds a specific substrate, such as carbohydrate in the cell wall of target bacteria [10]. The catalytic VanY domain is conserved in other L-alanoyl-D-glutamate endopeptidases, including CwlK in *B. subtilis *and Ply500 in *L. monocytogenes *bacteriophage A500 [23,25] and D-alanoyl-D-alanine carboxypeptidases [26]. The SH3_5 domain at the C-terminus was found in the putative lysins of *Bacillus *bacterial strains, *Bacillus *phages and *Lactobacillus *phages (Figure 1a), suggesting that this domain is the cell wall binding domain.

Biochemical characterization showed that the LysB4 endolysin was slightly alkalophilic, because activity was optimal at pH 8.0-10.0. It was also slightly thermophilic, with an optimal temperature of 50°C. The maximal lytic activity occurred in the absence of NaCl. This enzyme required a divalent metal ion, such as Zn^2+ ^or Mn^2+^, for full enzymatic activity. A similar requirement for divalent cations was seen for Ply500 in *L. monocytogenes *bacteriophage A500 [23]. The other characterized L-alanoyl-D-glutamate peptidase, T5 endolysin requires Ca^2+ ^instead of Zn^2+ ^or Mn^2+ ^[24]. The requirement of Zn^2+ ^or Mn^2+ ^is supported by protein sequence analysis, because LysB4 has the three Zn^2+^-coordinating residues (His80, Asp87, His133) of Ply500, and the Zn^2+-^binding domain (SxHxxGxAxD) [22].

Endolysins are generally known to be highly specific against particular species of bacteria. However, LysB4 showed lytic activity against a broad range of bacterial species. LysB4 showed similar activity toward susceptible Gram-positive and Gram-negative bacteria, whereas other reported L-alanoyl-D-glutamate endopeptidases have a much narrower target host range [23]. LysB4 could lyse not only *B. cereus *strains but also other Gram-positive bacteria such as *B. subtilis *and *L. monocytogenes *strains. In addition, this enzyme also showed lytic activity toward Gram-negative bacteria when treated with EDTA. Most Gram-negative bacteria contain the Alγ type peptidoglycan, and *Bacillus *species and *L. monocytogenes *have the Alγ type cell wall as well [23,24,27,28]. Thus, LysB4 probably targets Alγ type peptidoglycan. This relatively broad antibacterial spectrum of LysB4 was surprising, given the narrow host range of the bacteriophage B4. Bacteriophage B4 only targets one strain of *B. cereus *(strain ATCC 10876) of five tested *B. cereus *strains and other Gram-positive bacterial species including *L. monocytogenes *strains, *S. aureus*, and *Ent. faecalis *(Shin *et al. *unpublished). This suggests that there are more bacterial species with the LysB4 cell wall recognition site than those containing the bacteriophage B4 receptor. Therefore, further studies are needed to determine the moiety targeted by the LysB4 cell-wall binding SH3_5 domain.

## Conclusions

LysB4 is the first characterized L-alanoyl-D-glutamate endopeptidase originating from a *B. cereus *bacteriophage. Although LysB4 has similar enzymatic and genetic properties to Ply500 from *L. monocytogenes *bacteriophage, LysB4 has broader spectrum and can lyse both Gram-positive and Gram-negative bacteria, including a number of foodborne pathogens. As this enzyme also shows strong lytic activity and stability in wide range of pH and temperature, LysB4 has high potential as an effective antibacterial agent to control foodborne pathogens. In the presence of agents such as EDTA, which permeabilize the outer cell membrane [29], LysB4 could be successfully applied exogenously to control Gram-negative bacteria as well as Gram-positive bacterial pathogens.

## Methods

### Bacterial strains, phage and growth conditions

*B. cereus *ATCC 10876 was used as the host for bacteriophage B4 (KCTC 12013BP) and the substrate for the LysB4 endolysin. *E. coli *BL21 (DE3) was used as the host for expression of the recombinant LysB4. Bacterial strains that were used for antimicrobial spectrum determination are described in Table 2 along with the results. All the bacterial strains were routinely grown at 37°C in Luria-Bertani (LB) broth medium (Difco). Ampicillin (50 μg/ml) was added when necessary.

### Cloning, expression, and purification of LysB4

The endolysin gene (*lysB4*) was amplified from the genomic DNA of the bacteriophage B4 by polymerase chain reaction (PCR) using primers lysB4F (5'-AGTGGAAGTCATATGGCAATGGCATTA-3') and lysB4R (5'-TAAAAAAAGGATCCCCGAAGGACTTCC). The PCR product was cloned into pET15b (Novagen), which has an N-terminal hexahistidine (His)-tag sequence. The correctly cloned plasmid was transformed into competent *E. coli *BL21 (DE3). Expression of the recombinant LysB4 was induced with 0.1 mM isopropyl-β-D-thiogalactopyranoside at OD_600 _1.0, followed by incubation for an additional 5 h at 30°C. Bacterial cells were suspended in lysis buffer (50 mM potassium phosphate, 200 mM sodium chloride, pH 7.0) and disrupted by sonication (Branson Ultrasonics). After centrifugation at 15,000 × *g *for 20 min, the supernatant was passed through a Ni-NTA Superflow column (Qiagen), and purification of the recombinant LysB4 was performed according to the manufacturer's instructions. The purified protein was stored at -80°C until use after the buffer was changed to the storage buffer (50 mM potassium phosphate, pH 8.0, 200 mM NaCl, 30% glycerol) using PD Miditrap G-25 (GE Healthcare).

### Lytic activity assay

The lytic activity of the endolysin against bacterial cells was assayed by monitoring the decrease in OD_600 _[30]. *B. cereus *ATCC 10876 or other bacteria were cultivated to exponential phase. Cells were harvested and resuspended with the reaction buffer (50 mM Tris-HCl, pH 8.0) to adjust OD_600 _to 0.8-1.0. When needed, 0.1 M EDTA was used to treat the Gram-negative bacteria after harvesting, as described previously [31]. The endolysin (100 μl) was added to the cell suspension (900 μl) followed by incubation at room temperature, unless indicated otherwise. OD_600 _values were monitored over time. The lytic activity was calculated after 5 min as followed; {ΔOD_600 _test (endolysin added) - ΔOD_600 _control (buffer only)}/initial OD_600._

To evaluate the effect of pH on LysB4 enzymatic activity, the endolysin (5 μg) was added to *B. cereus *cells suspended with a variety of buffers: 0.1% trifluoroacetic acid (TFA) for pH 2.0; 50 mM sodium acetate for pH 4.3; 50 mM 2-(N-morpholino)ethanesulfonic acid for pH 6.0; 50 mM Bis-Tris for pH 7.0; 50 mM Tris-HCl for pH 8.0-8.5; 50 mM glycine for pH 9.0-9.5; and 50 mM *N*-cyclohexyl-3-aminopropanesulfonic acid for pH 10.0-10.5. Different temperatures (25-72°C) were applied to test the effect of temperature on LysB4 (0.1 μg) enzymatic activity. To evaluate the stability of the endolysin, the lysis assays were performed against *B. cereus *ATCC 10876 at room temperature and pH 8.0 after the enzyme was incubated for 30 min under the selected pH conditions or at different temperatures. The influence of NaCl on lytic activity of LysB4 (1 μg) was tested with addition of various concentrations of 0, 50, 100, 150 and 200 mM NaCl.

The effects of metal ions on the lysis activity were determined as previously reported [32]. To chelate metal ions attached to the endolysin, EDTA (5.0 mM) was added to the enzyme (5 μg) and incubated at 37°C for 1 h. EDTA was removed by exchanging the buffer to reaction buffer using PD trap G-25 (GE Healthcare). The EDTA-treated enzyme was added to the cell resuspension with metal ions (ZnCl_2_, MgCl_2_, MnCl_2_, CuCl_2_, HgCl_2 _or CaCl_2 _0.1 or 1.0 mM) and the lysis activity was assayed in the reaction buffer.

### Assays for endopeptidases, glycosidases, and amidases

Endopeptidase activity was measured by quantification of liberated free amino groups from the peptidoglycan by the endolysin reaction. A crude cell wall of *B. cereus *was prepared by the method described by Kuroda and Sekiguchi [33], and to block pre-existing free amino groups in the peptidoglycan, *B. cereus *cell wall was acetylated as described by Pritchard *et al. *[34]. Free amino groups generated by digestion of the cell wall by LysB4 endolysin were assayed by the TNBS method [35]. Serine was used as the standard [36]. Glycosidase activity was confirmed by the method of Pritchard *et al. *[34] and amidase assay was performed as described by Hazenberg *et al. *[37].

### Determination of the cleavage site in peptidoglycan

The LysB4 cleavage site in the peptidoglycan was determined as described by Fukushima *et al. *[28]. Briefly, the acetylated peptidoglycan was digested with LysB4 for 0 and 60 min, and the released free amino groups detected by addition of 1-fluoro-2,4-dinitrobenzene, which forms 2,4-dinitrophenol (DNP) amino acid derivatives. These mixtures were hydrolyzed with 4 N HCl for 12 h at 97°C to digest glycosidic and peptide bonds. The DNP-labeled compounds were separated by RP-HPLC (HP1100) with Vydac C18 column (4.6 × 250 mm), using 365 nm for detection of the eluted products. Using two elution buffers (A, 0.025% TFA in water; B, 0.025% TFA in acetonitrile), elution was performed with a linear gradient of buffer B (0-100%) for 60 min at 40°C. After identifying the peaks, LC-MS analysis was performed to confirm the molecular mass of the peaks using Finnigan TSQ Quantum Ultra EMR (Thermo Scientific). This experiment was performed by Korea Basic Science Institute, Seoul Center (Seoul, Republic of Korea).

### Nucleotide sequence accession number

The nucleotide sequence of *lysB4 *was deposited to GenBank under the accession number JN616385.

## Authors' contributions

BS, JL and SR designed the study. BS performed the experiments. HS carried out the sequence analysis. BS, JY, and SR analyzed the data and wrote the paper. SH critically reviewed the manuscript. All authors read and approved the final manuscript.
